# A patient journey map based on the experience of temporomandibular disorders patients: a qualitative systematic review and meta-synthesis

**DOI:** 10.3389/fpubh.2026.1769781

**Published:** 2026-02-12

**Authors:** Min Huang, ShiNi Huang, Hui Ma

**Affiliations:** 1 Department of Basic Medicine, Chengdu University of Traditional Chinese Medicine, Chengdu, Sichuan, China; 2 Department of Nursing, Chengdu University of Traditional Chinese Medicine, Chengdu, Sichuan, China

**Keywords:** temporomandibular disorders, disorders, patient journey map, meta-synthesis, qualitative research, systematic review

## Abstract

**Background:**

The traditional nursing model struggles to meet the needs of patients with complex diseases such as temporomandibular disorders. Clinically, nurses should provide comprehensive and evidence-based services tailored to patient’s unique characteristics.

**Aims:**

By integrating qualitative evidence from patients with temporomandibular disorders, the journey map identifies needs and serves as a reference for optimizing care.

**Methods:**

The search scope covers the period from the inception of each database to March 2025, and 11 databases were systematically searched. Focused on collecting qualitative research literature concerning the experience of temporomandibular disorders patients. We integrated the research results by employing the Joanna Briggs’ meta-aggregation approach.

**Results:**

A total of 19 studies were included and categorized into four stages of the patient journey, resulting in four main themes that reflect the multidimensional healthcare needs of TMD patients. These themes are further broken down into 34 sub-themes across three dimensions: tasks, emotions, and pain points. The resulting patient journey map visualizes these stages and highlights specific needs at each point in the journey.

**Conclusion:**

The journey for TMD patients is long and complex, with evolving, multidimensional needs. Caregivers should help patients adjust psychologically and manage challenges by offering targeted information and support throughout their disease journey.

**Systematic Review Registration:**

https://www.crd.york.ac.uk/PROSPERO/view/CRD420251010686, identifier PROSPERO (CRD420251010686).

## 1 Introduction

Temporomandibular Disorders (TMD) are a group of conditions that affect the structure and function of the temporomandibular joint and its associated muscles, ligaments, and surrounding tissues (1). Primary manifestations include localized pain, jaw dysfunction, clicking or popping sounds, and limited mouth opening. These symptoms can impair daily activities such as eating and speaking, significantly reducing quality of life (2).

The etiology of TMD is multifactorial, involving biological, psychological, and social environmental factors, but the exact causes of TMD remain completely elucidated (3). Current research classifies the etiological factors of TMD into three distinct categories: predisposing, initiating, and perpetuating factors, which collectively drive disease onset and progression, leading to its characteristic chronic pain. Initiating factors include macro- or micro-trauma and abnormal loading of the masticatory system, while perpetuating factors encompass oral habits, emotional disorders, and social influences (4). Predisposing factors represent the individual’s inherent psychological or pathophysiological foundation, creating a favorable environment for TMD development (5).

TMD represents a significant public health concern; prevalence studies indicate that TMD affects 5–12% of the global population (6). The prevalence among women is higher than that among men, and this gender difference is believed to be possibly caused by the difference in estrogen levels. Estrogen can alter the laxity of ligaments and promote inflammatory responses, thereby exacerbating the instability of the temporomandibular joint in female patients (7). The prevalence and incidence of TMD pain increase with age, reaching a peak in middle age (8), and the pain pattern usually fluctuates over time (9).

Patient Journey Map (PJM) originated from the concept of “journey mapping” in market and service industry research. It serves as a tool for exploring product usage experiences. In recent years, due to its ability to integrate complex data and visualize cross-scenario events, PJM has gained recognition in the field of chronic disease care (10). PJM is a “patient-oriented” research method, with core elements including the timeline, service process, patient behavior, demand touchpoints, and emotional experience. By recording and creating illustrated maps, it measures and visualizes the individual’s experience throughout the medical service process, helping researchers understand the interaction between patients and the complex medical system and improve their experience (11). This approach does not focus solely on individual medical events or care behaviors, but rather helps researchers better understand how individuals enter, experience, and exit healthcare services by closely observing the entire process they undergo (12).

Qualitative interviews typically delve deeply into patients’ feelings, thoughts, and experiences throughout their healthcare journey. This includes the process of disease recognition and progression, discrepancies between pre-treatment expectations and actual care experiences, choices regarding treatment and care modalities, critical periods of psychological fluctuation and corresponding events, satisfaction with current care, and suggestions for future healthcare, etc. (13). In summary, this study employs qualitative research as the primary data collection method for patient journey map (14). Through meta-synthesis, it systematically retrieves global qualitative studies on TMD patient experiences, aggregating, refining, and analyzing fragmented subjective data to synthesize more universal and comprehensive conclusions. This provides theoretical foundations for optimizing the healthcare experience of TMD patients.

## 2 Methods

### 2.1 The guiding principles of methodology

Meta-aggregation is the process of analyzing, classifying, and summarizing the results of qualitative research during the systematic review of qualitative research (15). By integrating multiple qualitative studies’ outcomes, novel concepts are engendered and endowed with new interpretations and integrated significances (16). This study adopted the JBI meta-aggregation procedure, grounded in pragmatism and Husserl’s transcendental phenomenology, to guide data extraction and synthesis, generate comprehensive findings that can inform healthcare practice or policy, and guide practitioners and decision-makers (17–19). The study adopted the JBI qualitative research quality assessment criteria to appraise the quality of the included literature and followed PRISMA (Supplementary file S1) and ENTREQ (Supplementary file S2) guidelines for reporting. We registered the research protocol on PROSPERO(CRD420251010686).

### 2.2 Search strategy

Computerized retrieval was performed in Web of Science, PubMed, CINAHL, Cochrane Library, Embase, PsycINFO, SCOPUS, CNKI, CBM, Wan fang, and VIP for relevant qualitative studies regarding the disease journey of patients with temporomandibular disorders, with the retrieval time frame spanning from the inception of each database to March 2025. The search strategy includes terms such as “temporomandibular disorders,” “temporomandibular joint disorder,” “TMD,” “experience,” “emotion,” “needs,” “challenge,” “qualitative research,” “interview,” and “focus groups.” We present additional details of the search strategy in File S3.

### 2.3 Eligibility and screening of the literature

Based on the research requirements, we established this study’s inclusion and exclusion criteria. Here are the inclusion criteria:

(1) Participants (P): patients diagnosed with TMD without grave mental illness, irrespective of gender, treatment modalities, or disease stages.
(2) Phenomenon of interest (I): the journey of TMD patients, including experiences related to TMD in daily life or during treatment.
(3) Context (Co): patients diagnosed with TMD in hospital settings, home healthcare environments, or medical institutions.
(4) Study design (S): Qualitative research and mixed-methods research involving qualitative result reports are both admissible, with no constraints imposed on research approaches. In the context of mixed methods research, we analyze only the qualitative component.

Here are the listed exclusion criteria:

(1) Full-text literature that is inaccessible.
(2) Unofficially published theses or grey literature published in newspapers.
(3) Studies that report only quantitative or mixed results.

We used EndNote 21 to import all references and eliminate duplicates for the literature screening and analysis. Two researchers independently conducted the literature screening and data extraction process. We performed an initial screening of titles and abstracts to exclude irrelevant studies. Then, we reviewed the full texts to identify the eligible literature. Lastly, we cross-verified their findings to ensure consistency. A third reviewer resolved any divergences. For articles that meet the inclusion criteria, a second review is conducted following the process of the first round. The outcome of the literature screening is presented in the PRISMA flowchart.

### 2.4 Quality appraisal

All reviewers have received training in the methodology of evidence-based practice. Two reviewers independently evaluated the quality of the included studies using the JBI Qualitative Research Assessment Criteria. We determined each standard answer for the evaluation criteria as “yes,” “no,” “unclear, “or” not applicable” and classified the included studies into a tripartite grading system: “A,” “B, “and “C.” Completely meeting the aforementioned quality criteria, the possibility of bias was relatively low, and the quality level was classified as Grade A; Those partially complied with the aforementioned quality criteria and had a moderate likelihood of bias were classified as a quality grade of B; Those that were in total non-compliance with the above standards, with a considerable possibility of bias, were assigned a quality grade of C. Discussions will be conducted with three reviewers in the case of disagreement between two reviewers until a consensus is achieved. Ultimately, only studies with quality ratings of A and B were included in the research.

### 2.5 Data extraction

Two reviewers separately extracted data from the incorporated studies. We discussed any discrepancies with a third reviewer to resolve them, ensuring consensus among all reviewers. Standardized tables were used to extract the basic characteristics of the studies, including the author and publication year, country, study design, sample size, study subjects and their characteristics, phenomenon of interest, and the study’s main results.

### 2.6 Data synthesis and evidence assessment

This research utilized the JBI meta-aggregation methodology to synthesize the outcomes. At the beginning stage, reviewers conducted an exhaustive inspection of the included literature. They grouped them based on the findings of the included studies (themes and subthemes), then proceeded to classification. Ultimately, new “comprehensive discoveries” were generated from the categories, yielding comprehensive and representative conclusions. All the reviewers scrutinized the data analysis process and the comprehensive outcomes to guarantee the consistency of interpretation and the suitability of the analytical themes. Any divergences were settled through discussion. All analysis was performed in Excel.

For the integrated and synthesized evidence, two reviewers applied the ConQual approach to systematically assess the credibility and dependability of the evidence, yielding quality ratings of high, moderate, low, or very low according to the ConQual framework (20). Any discrepancies that arose during this process were resolved through discussion involving a third reviewer, ensuring consensus was achieved.

### 2.7 Construction of the journey map

Patient journey maps usually comprise a horizontal axis and a vertical axis (21).

#### 2.7.1 Horizontal axis

The horizontal axis represents the patient journey’s timeline or stages. Patients with chronic conditions have varying needs regarding disease status, information, and care at different stages (22). To provide targeted support, the first step in constructing a PJM is to segment and explore the entire healthcare process for patients at different disease stages (12).

#### 2.7.2 Vertical axis

The vertical axis generally includes the following five elements:

(1) Emotions: Researchers can analyze patients’ emotional trajectories by interviewing them, observing their behavior, facial expressions, and communication styles, and inquiring about their emotional experiences and reactions (23). By mapping the patient’s journey and drawing an emotional curve to reflect the patient’s emotional fluctuation points, emotional care support can be provided to increase the patient’s confidence in facing the disease (24).
(2) Tasks: Tasks refer to the specific actions patients undertake to meet their health needs at different stages of their illness, as well as the corresponding service-delivery actions of the medical system. These tasks span the entire patient journey, and the quality of their completion directly affects treatment outcomes and the medical experience (25).
(3) Pain points: Pain points refer to the obstacles, dissatisfaction, or unmet needs that patients encounter during the process of completing tasks and interacting with the medical system (26, 27).
(4) Touchpoints: A touchpoint refers to all the interaction nodes that occur between a patient and the medical service system and related parties throughout the entire medical journey, from the generation of health needs to the end of diagnosis and treatment and follow-up care, in order to achieve the goals of diagnosis, treatment, care, consultation, and service (28, 29).
(5) Opportunity points: Opportunity points are identified through pain point analysis, combined with advancements in medical technology and service model innovation (25). Research members first collected medical literature and clinical guidelines related to TMD in recent years through a desktop research system, and after discussion, initially drew the timeline of the patient journey map. Then, based on the qualitative research data included in the literature and combined with the patients’ self-descriptions in the data, they analyzed and extracted the time points and emotional expressions of the patients, as well as the questions they had and the types of help they sought, systematically extracting the basic elements of the patient journey map. Similar semantic elements were merged and classified. Finally, the JBI meta-synthesis method was adopted to integrate the research results for comparison and contrast.

## 3 Results

### 3.1 Literature search results

**Figure 1:**
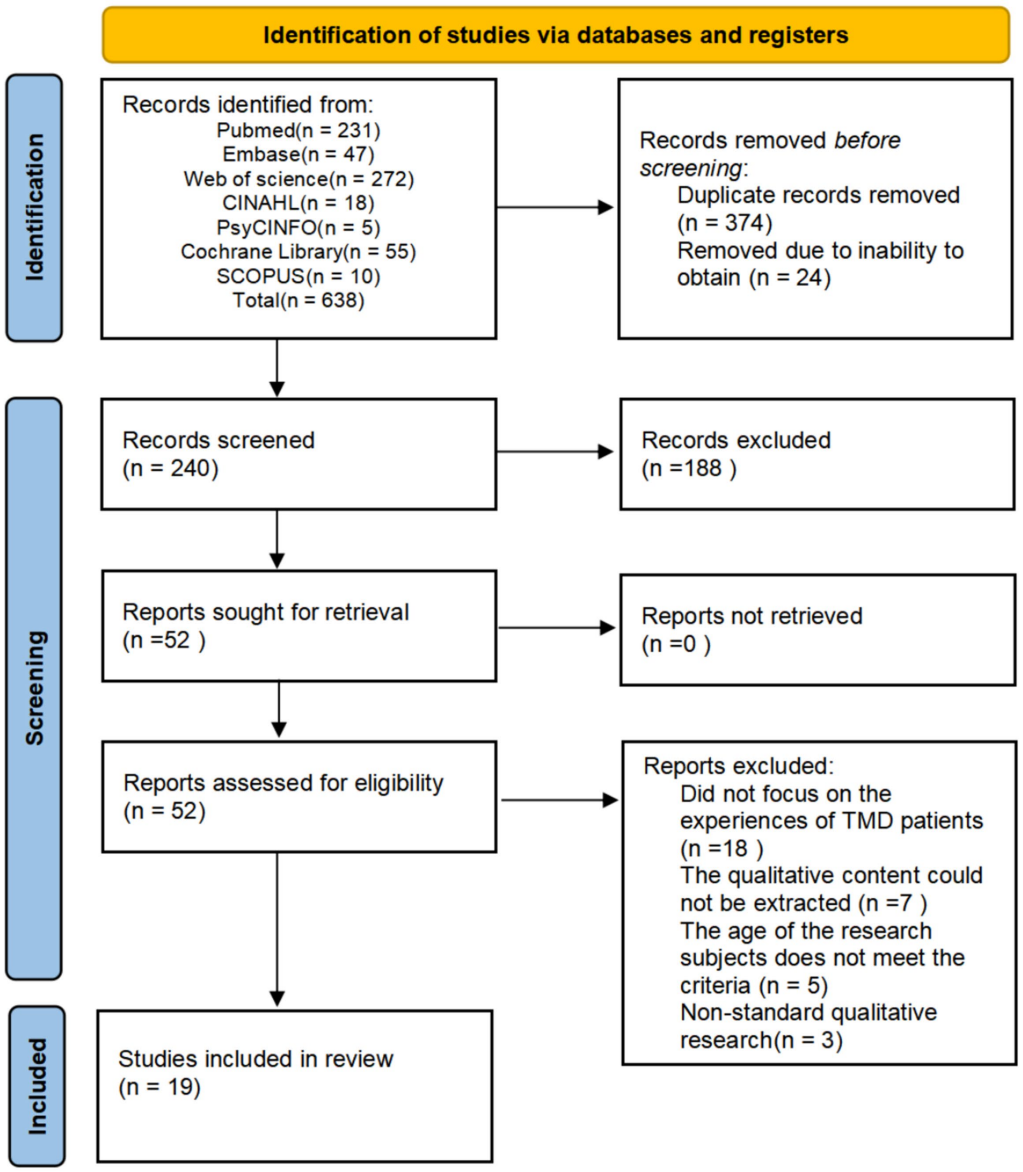
PRISMA flow diagram of literature screening.

Figure 1 shows the literature screening process. We retrieved 638 studies from databases. Then, using EndNote 21, we eliminated 24 studies that we could not obtain and removed 374 duplicate studies. After that, we reviewed the titles and abstracts according to the inclusion criteria and excluded 188 studies. Subsequently, we meticulously reviewed the full texts of the remaining 52 studies to further evaluate their compliance with the inclusion criteria. Finally, we included 19 articles (30–45).

### 3.2 Quality assessment

The quality assessment results of all included studies are supplied in Table 1 (Supplementary file S4). We graded all the studies as B. Among them, three studies did not clearly state whether the philosophical viewpoints and research methods were consistent, one study did not clearly state whether the research methods and data collection methods were consistent, and one study did not clearly state the consistency of research methods, data presentation, and analysis. Eight studies lacked clarity in their depictions of the researchers’ cultural background and values. At the same time, the remaining eight failed to offer detailed information on this aspect. Furthermore, two studies were ambiguous about whether they had an impact on the researchers, and this issue was not addressed in the other studies. All the studies received ethics committee approval.

**Table 1:** Quality appraisal of the included studies.

Author (Publication year)	Is there congruity between the stated philosophical perspective and the research methodology?	Is there congruity between the research methodology and the research question or objectives?	Is there congruity between the research methodology and the methods used to collect data?	Is there congruity between the research methodology and the representation and analysis of data?	Is there congruity between the research methodology and the interpretation of results?	Is there a statement locating the researcher culturally or theoretically?	Is the influence of the researcher on the research, and vice-versa, addressed?	Are participants, and their voices, adequately represented?	Is the research ethical according to current criteria or, for recent studies, is there evidence of ethical approval by an appropriate body?	Do the conclusions drawn in the research report flow from the analysis, or interpretation, of the data?
Garro (30)(1994)	Y	Y	Y	U	Y	N	N	Y	Y	Y
Durham et al. (31)(2010)	Y	Y	Y	Y	Y	N	N	Y	Y	Y
Durham et al. (32)(2011)	Y	Y	Y	Y	Y	U	N	Y	Y	Y
Rollman et al. (33)(2013)	Y	Y	Y	Y	Y	N	N	Y	Y	Y
Bonathan et al. (34)(2014)	Y	Y	U	Y	Y	N	N	Y	Y	Y
Mienna et al. (35)(2014)	Y	Y	Y	Y	Y	Y	N	Y	Y	Y
Eaves et al. (36)(2014)	Y	Y	Y	Y	Y	N	N	Y	Y	Y
Nilsson et al. (37) (2016)	U	Y	Y	Y	Y	Y	U	Y	Y	Y
Eaves et al. (38)(2017)	Y	Y	Y	Y	Y	U	N	Y	Y	Y
Fjellman-Wlklund et al. (39) (2019)	U	Y	Y	Y	Y	U	N	Y	Y	Y
Ilgunas et al. (40)(2020)	Y	Y	Y	Y	Y	U	N	Y	Y	Y
Dinsdale et al. (41)(2021)	U	Y	Y	Y	Y	Y	U	Y	Y	Y
Dinsdale et al. (42)(2021)	Y	Y	Y	Y	Y	U	N	Y	Y	Y
Taimeh et al. (43)(2022)	Y	Y	Y	Y	Y	N	N	Y	Y	Y
Ilgunas et al. (44)(2023)	Y	Y	Y	Y	Y	N	N	Y	Y	Y
Penlington et al. (45)(2024)	Y	Y	Y	Y	Y	N	N	Y	Y	Y
Baggen et al. (46)(2024)	Y	Y	Y	Y	Y	U	N	Y	Y	Y
Olsson et al. (27)(2024)	Y	Y	Y	Y	Y	U	N	Y	Y	Y
Safour et al. (47)(2024)	Y	Y	Y	Y	Y	U	N	Y	Y	Y

Y, Yes; N, No; U, Uncertain; NA, not applicable.

### 3.3 Basic features of literature

We present the characteristics of all included studies in Table 2 (Supplementary file S6). The period of the included studies ranges from 1994 to 2024. Among the 19 included studies, the sample size ranged from 6 to 300 participants, totaling 698 participants aged 15 to 74. Three studies failed to clearly specify the gender distribution, whereas in the remaining 16 studies, a total of 259 patients were involved, among whom 209 were female, and 50 were male. Detailed demographic information is provided in Table 3 (Supplementary file S9). Six studies (27, 35, 37, 39, 40, 44) were conducted in Sweden, while the others were carried out in different regions, including the Netherlands (33, 46), the United States (30, 36, 38), Australia (41, 42), the United Kingdom (31, 32, 34, 43, 45), and Canada (47). These investigations are mainly conducted in hospitals or dental clinics and rely on semi-structured interviews. One study employed random sampling; five used convenience sampling. Six studies adopted the purposive sampling approach, while other investigations recruited patients utilizing telephone or letter invitations, community publicity, and newspaper advertisements. Among them, 18 studies employed the phenomenological method, and 1 utilized the grounded theory.

**Table 2:** Characteristics of incorporated studies.

Author (Publication year)	Country	Research design (methodology and data collection method)	Sample size	Characteristics of participants	Phenomena of interest	Main research themes and subthemes
Garro (30) (1994)	USA	Phenomenological approach; Open-ended, semi-structured interview	32 (Female: 27; Male: 5)	The patient was diagnosed with temporomandibular joint disorder and reported a disease duration of at least 6 months, age range 23–69 years	Investigate the lived experiences of individuals diagnosed with TMD and describe the influence of this condition on their daily functioning and overall well-being.	1. The search for diagnosis
						2. The search for effective treatment
						3. TMJ: A problem or mind or body?
						4. Physicians are perceived to make similar attributions about TMJ
Durham et al. (31)(2010)	UK	Phenomenological approach; Semi-structured interviews	19 (Female: 14; Male: 5)	Patients with temporomandibular joint disorders who have undergone treatment at secondary-level medical institutions for a duration exceeding 3 months, age range 18–60 years	Describe the challenges faced by TMD patients in obtaining an accurate diagnosis and the resulting effects on their physical and psychological well-being.	1. Lack of diagnostic certainty
						2. Worry
						3. Repeatedly attended primary care
						4. Inappropriate and unsuccessful treatment
						5. Difficulty engaging professionals
						6. Questioning of the legitimacy of the complaint
						7. Diagnosis like therapy
Durham et al. (32)(2011)	UK	Phenomenological approach; Semi-structured interviews	29 (Female: 23; Male: 6)	Patients undergoing treatment for temporomandibular joint disorders with a treatment duration exceeding 3 months, age range 18–65 years	Investigate TMD patients’ experiences and their progression through the treatment process	1. Genesis of the problem
						2. Seeks help in primary care
						3. Secondary care intervention and TMDs’ negative impacts on everyday living
Rollman et al. (33)(2013)	Netherlands	Phenomenological approach; Semi-structured interviews	16 (Female: 12; Male: 4)	16 patients exhibiting TMD pain symptoms were recruited, with 8 participants (6 females and 2 males) seeking treatment and another 8 (6 females and 2 males) not seeking treatment. The two groups were well-matched in terms of age, gender distribution, pain intensity levels, and fear of movement	To explore the illness experience of patients with temporomandibular joint disorder (TMD)-related pain and assess potential differences between treatment-seeking and non-treatment-seeking individuals within this patient population	1. Person-related characteristics
						2. External circumstances
Bonathan et al. (34)(2014)	UK	Phenomenological approach; Semi-structured interviews	12 (Female: 9; Male: 3)	All patients experienced non-dental chronic orofacial pain, and the study involvement took place before their first visit to a specialized facial pain clinic, age range 26–73 years	Investigating the illness experience and perception of facial pain among TMD patients	1. Need for information to counteract helplessness
						2. Worry as part of the process of making sense of the pain
						3. Validation of the pain experience
						4. The importance of trust
Mienna et al. (35)(2014)	Sweden	Grounded Theory; Qualitative thematic interviews	17 (Female: 17)	Sami women, 10 individuals were diagnosed with TMD, whereas another 7 women of comparable age exhibited no signs or symptoms associated with TMD, age range 23–58 years	To investigate the perspectives, experiences, and beliefs of Sami women with and without TMD concerning the condition, along with their healthcare-related experiences	1. “Grin(d) and Bear it”
Eaves et al. (36)(2014)	USA	Phenomenological approach; Open-ended, semi-structured interview	44 (The original study did not provide information on the gender distribution)	All patients were diagnosed with TMD, reporting facial pain intensity exceeding 5 on the VAS at its peak severity (ranging from 0 to 10), and fulfilled at least one of the 10 predefined traditional Chinese medicine diagnostic criteria, age range 18–70 years	To investigated the illness experiences of TMD patients and examined the role of hope in their expectations, experiences, and evaluations of treatment outcomes	1. Realistic hope
						2. Utopian hope
						3. Wishful hope
						4. Technoscience hope
						5. Transcendent hope
Nilsson et al. (37) (2016)	Sweden	Descriptive phenomenological study; Semi-structured interviews	21 (Female: 19; Male: 2)	Teenagers with TMD pain, aged 15–19 years old	To investigate the illness experiences of adolescents, their interpretations of pain associated with TMD, strategies for managing such pain, and patterns of healthcare-seeking behavior.	1. Self-constructed explanation
						2. Pain management strategies
Eaves et al. (38)(2017)	USA	Phenomenological approach; Open-ended, semi-structured interview	95 (The gender distribution in the original study was unclear, but it was mainly composed of females)	Patients diagnosed with TMD and experiencing the most severe facial pain rated as 5 or higher on a 0-to-10 scale.	To explore TMD patients’ illness experiences before the intervention as well as throughout the entire treatment period.	1. The Work of Stoicism: “Just Dealing with Pain”
						2. Vigilance Work: Fear and the Work of Avoiding Triggers
						3. Refocusing on the Present: Vigilance against Biographical Disruption
Fjellman-Wlklund et al. (39) (2019)	Sweden	Phenomenological approach; structured interview	300 (The original study did not provide information on the gender distribution)	Adult patients diagnosed with TMD, aged 20 to 69 years old	To investigate the perspectives and experiences of these individuals regarding the management of their TMD symptoms	1. Being lost in dentistry
						2. No confidence in the dentists
						3. Expressions of both trust and belief
						4. Aware patients enable active coping
						5. Confirmation and information are appreciated
Ilgunas et al. (40)(2020)	Sweden	Phenomenological approach; Semi-structured interviews	9 (Female: 8; Male: 1)	All the patients suffered from TMD and were aware of their excessive joint mobility. Their ages ranged from 18 to 22 years old	To explore the experiences of young individuals in daily life concerning TMD, as well as their interactions with medical and dental care providers	1. Emotional perception
						2. Dealing with symptoms
						3. Outside influences
Dinsdale et al. (41)(2021)	Australia	Phenomenological approach; Semi-structured framework interviews	13 (Female: 12; Male: 1)	Patients diagnosed with TMD, aged from 22 to 61 years old	To investigate the management experiences, needs, and preferences of individuals with persistent intra-articular TMD	1. Searching for help
						2. Wanting answers
						3. Wanting to regain control
						4. Meeting needs, preferences and expectations, and the implications on care
Dinsdale et al. (42)(2021)	Australia	Phenomenological approach; Semi-structured interviews	16 (Female: 14; Male: 2)	All patients met the diagnostic criteria for intra-articular TMD and had experienced symptoms for at least 3 months. The average age of patients was 31.9 years	To explore the life experiences of adult patients with persistent intra-articular TMD, including the impact of TMD on activities, participation, and mental health, as well as the influence of environmental factors on disability.	1. The challenge of living with IA-TMD
						2. Living with uncertainty
						3. Seeking control
						4. Learning to live with it
Taimeh et al. (43)(2022)	UK	Phenomenological approach; Semi-structured interviews; focus groups	15 (Female: 14; Male: 1)	All participants were patients experiencing pain associated with TMD	To investigate patients’ experiences with TMD while accessing care through the National Health Service (NHS) in the UK and to identify their healthcare priorities when seeking treatment.	1. Access to appropriate care
						2. Organised and coordinated care
						3. Receving a diagnosis and enough information
						4. The interaction with the clinical staff
						5. Treatment strategies and having an ‘action’ plan
						6. Support and social networks
Ilgunas et al. (44)(2023)	Sweden	Phenomenological approach; Semi-structured interviews	16 (Female: 10; Male: 6)	All participants underwent evaluation based on the TMD diagnostic criteria and fulfilled at least one DC/TMD diagnostic criterion, with ages ranging from 22 to 65 years	To investigate the experiences of patients with TMD regarding diagnosis, treatment, and management, with a specific focus on living with TMD, factors influencing treatment-seeking behavior, and patients’ perspectives on the care received	1. Normal daily life despite aggravating circumstances
						2. Breaking point for seeking medical care depends on the manageability of discomfort
						3. Difficulties to receive the appropriate treatment
						4. Expectations on dental care providers normally not met
Penlington et al. (45)(2024)	UK	Phenomenological approach; Semi-structured interviews	21 (Female:13;male:8)	Patients diagnosed with persistent TMD, aged 19–72 years.	To investigate the disease experience and understanding of patients with TMD, as well as the extent to which their understanding is shaped by healthcare providers	1. “a medically focused journey to nowhere-participants’ frustrated attempts to find medical management that will end their pain”
						2. Is it Me?—participants questioning their role in persisting pain symptoms.
						3. Participants’ emerging development of a holistic understanding of their TMD pain
Baggen et al. (46)(2024)	Netherlands	Phenomenological approach; semi-structured in-depth interviews	10 (Female: 7; male:3)	Patients who have experienced chronic orofacial pain (OFP) for at least 3 years and received treatment across multiple professional institutions before being diagnosed with TMD-related pain by an orofacial pain specialist and subsequently receiving treatment, with ages ranging from 43 to 71 years	To investigate the diagnostic and treatment history of patients with chronic TMD pain, identify factors contributing to delayed TMD diagnosis and inadequate timely treatment, and explore patients’ perspectives and experiences regarding potential improvements in their diagnostic and therapeutic processes	1. Chronic OFP complaints before TMD-pain treatment
						2. History of treatments of chronic OFP before TMD-pain treatment
						3. Experiences with TMD-pain treatment
						4. Bruxism and other behaviors
						5. Chronic OFP complaints after TMD-pain treatment
						6. Patient’s perspective on improving chronic OFP care
Olsson et al. (27)(2024)	Sweden	Giorgi’s phenomenological method; semi-structured interviews	7 (Female: 6; Male: 1)	Patients with concurrent rheumatic inflammation and TMD, with ages ranging from 47 to 74 years	To investigate the experiences and perspectives of patients with rheumatic inflammation as they relate to TMD	1. Physical challenges of the jaw and a struggle to retain control
						2. Shame and social challenges
						3. Worrying about the future: frustration, grief, and a loss of freedom
						4. Defiance, endurance and efforts to maintain self-esteem
						5. health care experiences
Safour et al. (47)(2024)	Canada	Interpretative phenomenological approach; Semi-structured interviews	6 (Female: 4; Male: 2)	Participants had been diagnosed with TMDs for at least 6 months prior to the study and were aged between 25 and 64 years.	To achieve a comprehensive understanding of the life experiences of patients with chronic TMD, with a specific focus on how their condition influences their daily activities, such as dietary habits and social interactions.	1. Limited functioning and energy levels highlighting fatigue and frustration by TMD
						2. Communication challenges due to pain, affecting professional roles and social interactions
						3. Impact on social and professional life necessitating adjustments and accommodations
						4. Seeking medical help

**Table 3:** Demographic information of the participants.

Study ID	Year	Region	Sample size (*n*)	Age range (in years)	Gender ratio
Garro (30)	1994	USA	32	23–69	Female: 27; Male: 5
Durham et al. (31)	2010	UK	19	18 to 60	Female: 14; Male: 5
Durham et al. (32)	2011	UK	29	18 to 65	Female: 23; Male: 6
Rollman et al. (33)	2013	Netherlands	16	No information	Female: 12; Male: 4
Bonathan et al. (34)	2014	UK	12	26 to 73	Female: 9; Male: 3
Mienna et al. (35)	2014	Sweden	17	23 to 58	Female: 17; Male: 0
Eaves et al. (36)	2014	USA	44	18 to 70	No information
Nilsson et al. (37)	2016	Sweden	21	15 to 19	Female: 19; Male: 2
Eaves et al. (38)	2017	USA	95	No information	No information
Fjellman-Wlklund et al. (39)	2019	Sweden	300	20 to 69	No information
Ilgunas et al. (40)	2020	Sweden	9	18 to 22	Female: 8; Male: 1
Dinsdale et al. (41)	2021	Australia	13	22 to 61	Female: 12; Male: 1
Dinsdale et al. (42)	2021	Australia	16	No specific information (average age was 31.9)	Female: 14; Male: 2
Taimeh et al. (43)	2022	UK	15	No information	Female: 14; Male:1
Ilgunas et al. (44)	2023	Sweden	16	22 to 65	Female: 10; Male: 6
Penlington et al. (45)	2024	UK	21	19 to 72	Female: 13; Male: 8
Baggen et al. (46)	2024	Netherlands	10	43 to 71	Female: 7; Male: 3
Olsson et al. (27)	2024	Sweden	7	47 to 74	Female: 6; Male: 1
Safour et al. (47)	2024	Canada	6	25 to 64	Female: 4; Male: 2

### 3.4 ConQual assessment to gauge the strength of evidence

The ConQual system scores for this study are presented in File S5. The reliability scores of all the included studies were mostly 3 points. Each evidence level in the qualitative research was explicit, with the credibility remaining unaltered. Eventually, we affirmed that the ConQual level was moderate.

### 3.5 Patient journey map

**Figure 2:**
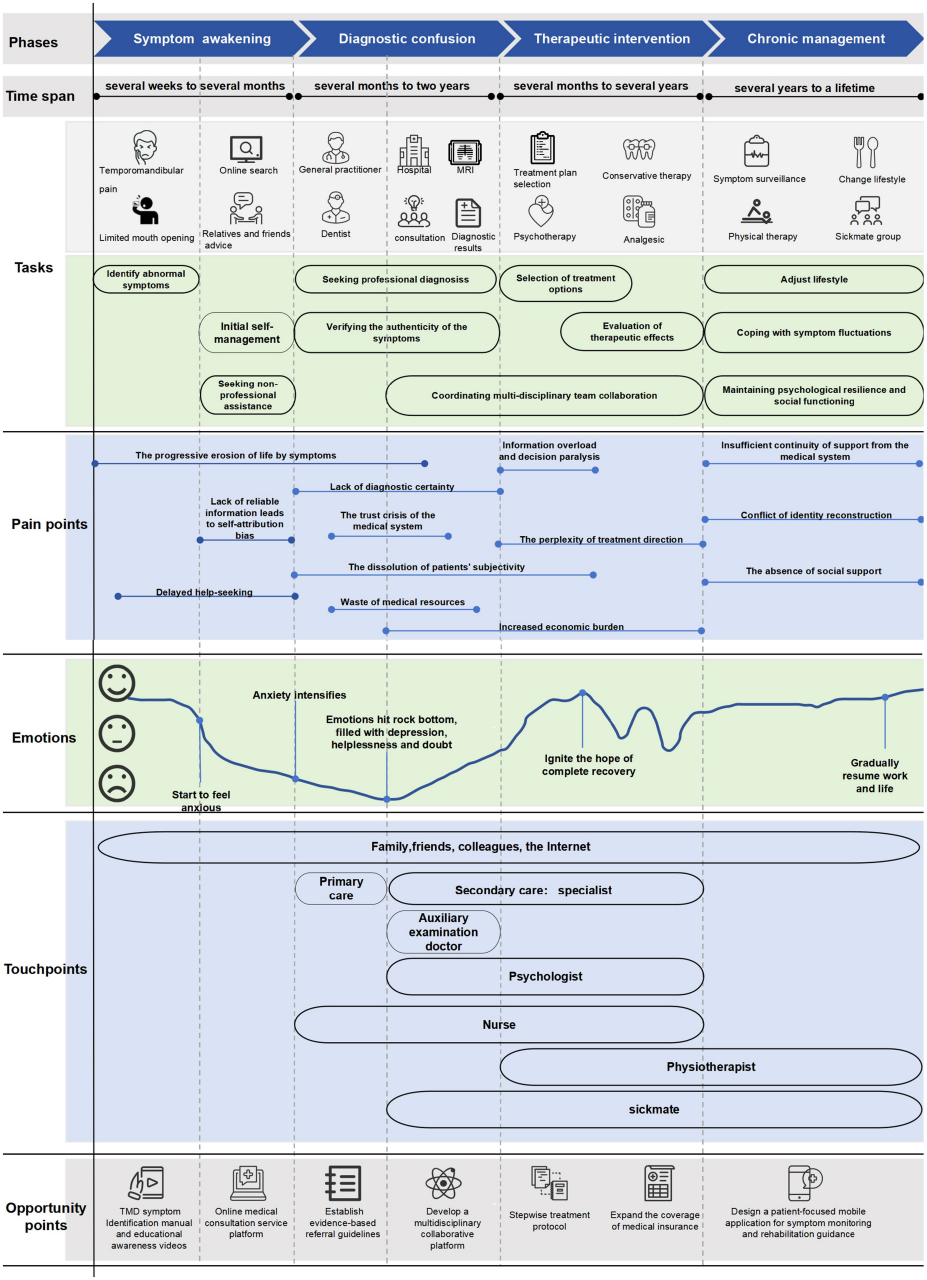
TMD patient journey map.

In summary, the findings were divided into four phases along the horizontal axis, yielding four comprehensive research outcomes. Additionally, 34 sub-themes were extracted from the three dimensions of tasks, emotions, and pain points along the vertical axis. All extracted themes are presented in Table 4 (Supplementary file S8). Based on the research findings, a patient journey map for TMD patients was drawn, as shown in Figure 2 (Supplementary file S7).

**Table 4:** Synthesized research themes.

Phase	Tasks	Emotions	Pain points
Symptom awakening onset of symptoms and confusion in self-management	Identify abnormal symptoms	Anxiety	The progressive erosion of life by symptoms
	Initial self-management	Worry	Lack of reliable information leads to self-attribution bias
	Seeking non-professional assistance		Delayed help-seeking
Diagnostic confusion: diagnostic uncertainty and the dilemma of medical interaction	Seeking professional diagnosis	Anxiety intensifies	Lack of diagnostic certainty
	Verifying the authenticity of symptoms	Doubt	The trust crisis of the medical system
		Depression and helplessness	The dissolution of patients’ subjectivity
			Waste of medical resources
Therapeutic intervention: the trade-offs and uncertainty of treatment options	Selection of treatment options	Hope and disappointment intertwined	Information overload and decision paralysis
	Evaluation of therapeutic effects	Fear	The perplexity of treatment direction
	Coordinating multi-disciplinary team collaboration		Increased economic burden
Chronic management: the long-term adaptation and the reconstruction of quality of life	Adjust lifestyle	Acceptance	Insufficient continuity of support from the medical system
	Coping with symptom fluctuations	Fatigue	Conflict of identity reconstruction
	Maintaining psychological resilience and social functioning	Loneliness	The absence of social support

#### 3.5.1 Symptom awakening phase: onset of symptoms and confusion in self-management

After patients perceive abnormal signals such as pain and clicking in TMJ, they undergo a progressive process of “self-attribution, initial self-management, and delayed medical treatment.” They use self-management strategies, such as adjusting their diet and self-massaging, to alleviate muscle tension. However, they often fall into attribution errors due to a lack of information or choose to ignore symptoms due to their concealed nature. During this phase, patients mainly rely on recommendations from relatives and friends, and online searches are their primary source of information. Nevertheless, unprofessional guidance exacerbates anxiety and confusion, leading patients to waver between “endurance” and “seeking medical treatment” until the pain and functional disorders surpass the psychological tolerance threshold before seeking medical assistance.

##### 3.5.1.1 Tasks

At the initial stage of symptom onset, patients primarily focus on identifying abnormal symptoms, implementing preliminary self-management, and seeking non-professional assistance. Research indicates that the earliest symptoms patients typically notice involve the mandible, such as pain in the temporomandibular region, limited mouth opening, and joint clicking. However, these symptoms are often mistakenly attributed to incidental factors, such as “growing pains,” or simply ignored in the early stages (27, 32, 35, 40). As symptoms persist, patients gradually recognize the potential nature of their disease and adopt various self-management strategies. These include physical interventions (such as massage and exercises), behavioral adaptations (such as limiting mouth opening and adjusting eating habits), and psychological coping mechanisms (such as ignoring pain or regulating stress). Some patients also develop non-medical etiological beliefs based on personal experience (e.g., analogies to joint dislocation) (27, 32–34, 38, 40, 44). Patients commonly rely on non-professional sources such as friends’ experiences or online searches to explain symptoms, validate personal perceptions, and set expectations (33, 39, 40, 44, 46).

*“It is making noise because I am growing. But now……I was cracking more than everyone else.”* (40).

*“To check if my complaint is something serious, I use the Internet, talk to friends, but I do not go to my general practitioner.”* (33).

##### 3.5.1.2 Emotions

Patients generally have anxiety and worry. Anxiety manifests as fear of losing control over the course of the disease and over-interpreting somatic symptoms, often leading to negative thoughts such as “my symptoms are more serious than imagined” and “my body is broken” (27, 32, 40, 44). Worry centers on the shadow of genetic risk or excessive imagination of disease severity (27, 33, 40, 42).

*“There’s a little bit of fear I think when something is more wrong than usual. Now something has broken.”* (44).

##### 3.5.1.3 Pain points

TMD symptoms progressively erode patients’ quality of life, evolving from intermittent discomfort to persistent distress, leading to functional decompensation in the orofacial region, following an evolution trajectory from “tolerable to uncontrollable” (27, 30, 32, 34, 42, 44). At the same time, patients often exhibit self-attribution bias due to limited medical knowledge, information overload from online sources, and insufficient ability to filter information. This may stem from unreliable online information, the belief that symptoms are linked to family genetics, or the direct association of stress with somatization symptoms (32, 35, 37, 38, 40).

*“It hurt so much just to eat normal foods.”* (44).

#### 3.5.2 Diagnostic confusion phase: diagnostic uncertainty and the dilemma of medical interaction

Upon entering the medical system, patients fall into a “referral maelstrom,” repeatedly shuffled among general practitioners, dentists, and specialists. Diagnostic uncertainty triggers a trust crisis. For example, symptoms are dismissed as “psychogenic” due to a lack of organic evidence, and multiple fruitless examinations result in the collapse of self-identification. The lack of cross-departmental collaboration subjects patients to the financial burden of repeated examinations, and some are compelled to suspend medical treatment due to stigmatizing labels. Anxiety and doubt intertwine, with patients having to contend with physical pain while also struggling to prove the authenticity of their symptoms within the healthcare system.

##### 3.5.2.1 Tasks

The core demand of patients at this stage is to seek professional diagnosis and validation of symptom authenticity. Patients urgently seek systematic diagnosis from medical authorities, desiring a definitive diagnosis at the initial consultation. However, they often require cross-departmental referrals, and multidisciplinary consultations at secondary medical institutions can enhance their confidence in treatment. A confirmed diagnosis not only provides medical justification for chronic pain but also grants patients social recognition (31–34, 41, 46). Simultaneously, patients frequently question the authenticity of their symptoms. Due to the absence of visible pathological evidence, their pain is often attributed to psychosomatic factors by others. Patients themselves fear falling into cognitive distortions of exaggerating their pain. Consequently, they seek medical authority to confirm the reality of their condition, striving to secure a genuine “patient identity” (30, 32, 34, 41, 43).

*“I feel I have a legitimate complaint, that it’s something that’s not in my head. I know there is a physical reason for it.*” (30).

##### 3.5.2.2 Emotions

The diagnostic dilemma faced by TMD patients manifests as a progressive psychological collapse characterized by escalating anxiety, doubt, frustration, and helplessness. Intensified anxiety manifests as catastrophic thinking about pain, such as associating it with cancer, worrying about worsening conditions, and heightened distress due to inefficient healthcare systems (e.g., prolonged waiting times, referral errors) (31, 32, 34, 43). This further leads patients into a cycle of mutual suspicion, where they not only self-negate and feel “ashamed of feigning illness,” but also question the professionalism of the medical system and doctors (30, 31, 35). Nine studies (30, 32, 33, 38–41, 43, 45) emphasize that the neglect of the medical system breeds depression and helplessness, that is, the repeated referral and shirking, as well as the psychological attribution of symptoms, deny the subjective experience of patients, deepening their feeling of “pain not being seen,” and ultimately leading to helplessness.

*“If the medical profession cannot put the finger on what’s wrong, they say it is in your head and send you to a shrink.”* (30).

##### 3.5.2.3 Pain points

TMD patients face four major challenges during diagnosis. First, the diagnosis lacks certainty (27, 30–34, 41, 43, 45), as its etiology involves multiple dimensions, and the clinical manifestations are heterogeneous, making it difficult for routine examinations to provide an accurate diagnosis. Moreover, due to insufficient cross-departmental collaboration and fragmented perspectives across disciplines, confusion persists regarding diagnostic labels. Additionally, patients’ descriptions of pain and their subjective experiences of functional limitations often do not match the objective examination results, further exacerbating the cognitive gap between doctors and patients. These factors collectively form a “diagnostic fog,” forcing patients to repeatedly seek verification within the medical system. The second is that the patient’s subjectivity gradually dissolves amid physiological loss of control, psychological self-criticism (e.g., equating clenching the teeth with a hot temper), and social stigmatization (being labeled a “difficult patient”) (27, 30–32, 40, 43). The third issue is that patients have a crisis of trust in the medical system, which is manifested in institutional indifference, such as their demands being ignored or not feeling listened to and understood after communicating with professionals, poor doctor-patient communication, and doubts about the professionalism of medical staff (30, 32, 34, 35, 39, 40, 44). The fourth issue is the waste of medical resources. Due to systemic inefficiencies in the referral process and repetitive consumption during diagnosis and treatment, medical resources are wasted. That is, the energy of the medical system that should be used for precise treatment is consumed in a meaningless process of idling (31, 33, 43).

*“…they started sending me again and again…then I came to the rheumatologist, and they started to look at it from another perspective, and she sent me to the orofacial pain specialist.”* (27).

*“After mentioning my complaints to my dentist, it still took at least half a year before she pointed out this clinic to me.”* (33).

#### 3.5.3 Therapeutic intervention phase: the trade-offs and uncertainty of treatment options

Patients struggle to make choices among fragmented treatment options, ranging from occlusal splints to botulinum toxin injections, from physical therapy to psychological intervention. The unpredictability of therapeutic effects triggers a “hope-disappointment cycle.” Communication barriers within multidisciplinary teams intensify decision-making paralysis, and some individuals voluntarily discontinue effective regimens due to economic burdens. Treatment failure not only induces physical suffering but also triggers the collapse of self-efficacy. They long to take control of their condition yet have to accept the reality of “living with the disease,” getting trapped in a dual predicament of treatment dependence and self-doubt.

##### 3.5.3.1 Tasks

During the treatment stage, the patient’s main tasks are to select a treatment plan, evaluate the therapeutic effect, and receive treatment from a multidisciplinary team. Seven studies (30, 33, 37, 41, 43, 44, 46) indicated that TMD treatment plans exhibit stepwise and individualized characteristics, with conservative approaches predominating. Patients’ treatment priorities are driven by symptoms, such as pain relief, and their expectations for treatment methods evolve dynamically, initially relying on medical intervention and gradually shifting toward self-management. When evaluating therapeutic effect, patients strongly desire to “regain control,” which involves not only relief of physical symptoms but also restoration of psychological and social functions. Moreover, for some patients, encountering good medical practitioners is of great significance (30, 42). TMD is caused by multiple factors, and single-discipline treatment has limitations. Only through the collaboration of a multidisciplinary team can an integrated treatment framework be constructed to provide patients with comprehensive and personalized plans, thereby more effectively assisting them in restoring their health (27, 35, 41, 43, 44, 46).

*“I found it [interprofessional treatment] very intensive, but I experienced it as pleasant.”* (41).

##### 3.5.3.2 Emotions

TMD patients experience profound emotional turmoil throughout their treatment journey. Eight studies (27, 30, 31, 34–36, 43, 45) reveal they remain trapped in a cycle of hope and disappointment: initially brimming with anticipation, their confidence is briefly restored by temporary symptom relief such as reduced pain. Yet when treatment fails to meet expectations, disappointment follows—only for them to persist in trying new therapies, reigniting hope. This cycle repeats endlessly. Concurrently, three studies (29, 31, 40) highlight that patients are also deeply entrenched in fear, both regarding treatment risks like invasive surgeries and the persistent shadow of symptom recurrence.

*“When you are pain free, how long are you going to go on this way before it flares up again… when is the next time?”* (30).

##### 3.5.3.3 Pain points

TMD patients face the dilemma of information overload and decision paralysis in treatment choices. The conflicting multidisciplinary treatment plans, such as the opposition between dental correction and psychological stress management, the conflicts among doctors’ opinions, and the imbalance of power between doctors and patients (“seemingly multiple choices but actually dominated by one party”), make it difficult for them to make effective choices (27, 30, 34, 41, 43, 45). The confusion about the treatment direction stems from the ambiguous treatment goals due to the chronic course of the disease, and the fragmentation of the medical system’s goals, where cure is simplified to short-term pain relief, forcing patients to struggle between physical repair and functional reconstruction (30, 31, 36, 41, 43, 45). Additionally, patients bear increased financial burdens due to high long-term treatment costs and inadequate insurance coverage (30, 38, 41, 44).

*“I did not feel like I needed to see a psychologist. I was pretty happy at that stage.”* (41).

#### 3.5.4 Chronic management phase: the long-term adaptation and the reconstruction of quality of life

Patients embark on a lifelong journey of chronic disease management, maintaining a delicate balance through dietary adjustments, stress management, and other daily behaviors. Periodic recurrences and functional limitations compel them to redefine their lives; that is, social activities decrease due to difficulties in eating, and career trajectories change due to fluctuating pain. The persistent lack of support from the healthcare system exacerbates feelings of isolation. Although most patients learn to reconcile with their symptoms, the long-term physical and psychological exhaustion leaves them exhausted and lonely, searching for meaning in life amid acceptance and resistance.

##### 3.5.4.1 Tasks

TMD patients employ multiple adaptive strategies during the management phase. The first step is to adjust their lifestyle by restricting their diet, controlling their behavior, and reconstructing social roles (such as choosing low-stress jobs) to avoid risks (30, 40, 42, 44). The second approach involves managing symptom fluctuations by utilizing a “risk warning” model, which includes self-treatment (such as over-the-counter drugs, stretching, and massage), behavior modification (correcting chewing habits), and immediate relief measures (physical therapy interventions) (35, 38, 42, 44, 46). Five studies (27, 32, 35, 38, 42, 44) also indicate that patients achieve psychological adaptation and social reintegration by maintaining psychological resilience and social function. On the basis of a clear diagnosis and trust in treatment, they rebuild themselves, conceal pain in social interactions, actively engage in daily activities, and minimize the excessive influence of the disease on their life and work.

*“I would yawn, and I would not open all the way because I would be apprehensive of it hurting.”* (42).

##### 3.5.4.2 Emotions

In the later stages of TMD, patients undergo a triple shift toward acceptance, fatigue, and isolation. Seven studies (27, 32, 34–36, 40, 42) show that TMD patients gradually accept the disease as they cope with it, regarding chronic symptoms and joint noises as normal. However, long-term symptom distress leads to reduced sleep quality and loss of appetite, resulting in fatigue. Moreover, due to functional limitations such as speaking and eating, patients face numerous obstacles in social interactions and are forced to reduce social activities, which intensifies their sense of loneliness.

*“It’s not the pain itself that’s worst, it’s everything around it, it’s the social part.”* (27).

##### 3.5.4.3 Pain points

Five studies (27, 43–46) have shown that continuous support from the medical system is insufficient, including frequent postponement or cancellation of check-up appointments and a lack of regular follow-up for many years, which fails to meet the needs of chronic disease management. The conflict caused by the disease in the reconstruction of the patient’s identity, the change in the way of action due to pain, and the limitation of speech that makes them alienated from normal social and occupational roles, the loss of the original quality of life and identity, and even pain becoming the core of self-identity (27, 30, 34, 38, 42, 47). Four other studies (32, 43, 45, 46) have noted that patients often lack social support. Insufficient understanding of the disease by relatives and friends leads to emotional isolation, and the pressure of seeking medical treatment is mostly borne by the patients themselves.

*“I have not had a routine check-up for several years……there have been postponed…canceled…appointments.”* (44).

*“Pain to me is something that’s debilitating in a sense. It changes the way that you move in the world.”* (38).

## 4 Discussion

This study utilized a visual patient journey map tool, with the four sequential stages as the horizontal axis and the three dimensions of tasks, emotions, and pain points as the vertical axis, to present the themes and sub-themes in an orderly manner. It also displayed the relevant personnel for the entire disease course’s health management and their coverage periods as touchpoints, to restore patients’ real experience throughout the entire disease course. Finally, based on the multidimensional cross-analysis of the horizontal and vertical axes, potential optimization solutions were provided.

### 4.1 Emotions drive actions

From the emotional curve plotted in the figure, we can see that emotional fluctuations experienced by patients with TMD during their disease journey are not only psychological experiences but also the core driving force behind their health behaviors and decisions. The anxiety and confusion during the symptom-awakening period led patients to rely on non-professional information sources for self-attribution, exacerbating delays in seeking medical treatment. The uncertainty during the diagnosis period triggers patients’ suspicion, which gradually evolves into deep frustration with the medical system (48). This “suspicion-frustration” emotional chain directly weakens the trust between doctors and patients, reflecting the impact of emotional experiences on the quality of medical interactions under the “biological-psychological-social” medical model. The coexistence of the “hope-disappointment” cycle during the treatment intervention period and the “acceptance-fatigue” during the chronic management period reveals that the shaping of behavior by emotions in the long-term disease course has phased characteristics.

Previous studies have confirmed that some psychological variables can affect the treatment compliance and therapeutic effect of patients with TMD. For instance, elevated anxiety levels may counteract the positive effects of physical treatments for TMD—such as bite splints and physical therapy—as well as medications designed to alleviate TMD pain (49). Stress is widely recognized as a common trigger of TMD symptoms (50), while cognitive-behavioral therapy can help individuals identify stressors and develop effective strategies to manage and reduce stress (2). Other research has found that antidepressants may help relieve pain and improve the function of patients with chronic TMD, especially when used in combination with other treatment methods (51).

Therefore, future research can further explore the application potential of emotional intervention strategies in the management of TMD. Especially in the treatment of TMD, more attention should be paid to the psychological state of patients, especially those with a longer course of disease. While alleviating clinical symptoms, more attention should be paid to the assessment of mental and psychological conditions. This not only improves patients’ psychological state but also positively impacts overall disease management (52).

### 4.2 Differences in touchpoints efficiency

The efficiency gap across medical touchpoints directly shapes patients’ diagnostic experiences and outcomes. Inefficiencies at primary care points are particularly evident, as general practitioners often lack sufficient awareness of TMD, resulting in misdiagnoses or delayed referrals that hinder treatment efficiency (53). Conversely, multidisciplinary collaboration points play a pivotal role in TMD diagnosis and treatment, enabling more precise diagnoses and effective therapies while enhancing patient confidence and treatment adherence (54). During chronic management phases, patients’ needs shift toward long-term support. However, the absence of sustained follow-up and support leads to poor self-management outcomes and increased susceptibility to disease recurrence (53, 55).

Differences in contact point efficiency provide guidance for optimizing nursing practice. Nursing staff should recognize the limitations of primary care touchpoints and the importance of multidisciplinary collaboration touchpoints, focusing on patients’ long-term support needs during chronic management. To optimize primary touchpoints, nursing staff can assist physicians in accurately assessing conditions and clarifying referral criteria by participating in the design and promotion of TMD symptom identification checklists, thereby improving the accuracy of initial diagnoses. Within multidisciplinary collaboration, nursing staff can transition from executors to coordinators. For instance, they can regularly convene professionals from departments such as dentistry, psychology, and rehabilitation to discuss complex TMD cases, develop integrated care plans, and enhance team effectiveness.

### 4.3 Upgrade path for pain points

The pain points of TMD patients are not isolated but rather form an escalating network of “individual-medical-social” interaction during the transition of disease stages. The self-attribution bias during the symptom-awakening phase and the delayed seeking of assistance, resulting in the chronicity of symptoms due to the absence of timely intervention, subsequently lead to multiple referral burdens and diagnostic uncertainties during the diagnostic stage. This escalation of pain points from information deficiency to institutional neglect is the superimposed effect of insufficient health literacy and the ambiguity of labor division in the medical system. The information overload and decision paralysis during the treatment and intervention period further disclose the collaborative flaws within the medical system. When there are conflicts in the explanations of disease causation across different departments, patients are compelled to bear the cognitive load of integrating across theoretical systems, and the imbalance of power between doctors and patients aggravates the sense of inability in decision-making. When the treatment effect is unsatisfactory, they doubt their ability to overcome the disease, which in turn influences their subsequent treatment enthusiasm. This escalation of pain points not only impacts treatment compliance but also results in the collapse of self-efficacy. Entering the chronic management phase, the cumulative effects of the early pain points are manifested as conflicts in identity reconstruction and the absence of social support. Due to the long-term restrictions imposed by the symptoms, patients gradually internalize the disease as the core of their self-identity, and their lifestyles, social activities, and even career advancement are profoundly affected (38). Moreover, the social cognitive biases regarding TMD cause patients to be stigmatized and experience a sense of isolation (56). The deficiency of support for chronic disease management within the medical system further exacerbates this isolated state.

The comprehensive management of TMD demands collaborative optimization among the medical system, individuals, and society. In terms of individual empowerment, by developing patient education tools such as disease self-assessment AI or disease knowledge bases, we aim to correct attribution biases in patients’ disease cognition, enhance their health literacy, and enable them to understand diseases and manage symptoms more scientifically (57). In terms of the medical system, we optimize cross-departmental collaboration mechanisms and clarify the division of responsibilities to reduce referral chaos and diagnostic uncertainty. On the other hand, a stepped treatment protocol should be implemented, gradually shifting from conservative treatment to invasive intervention, thereby reducing the decision-making burden for patients (58). At the social level, it is essential to enhance support for TMD patients, conduct public education campaigns to eliminate stigmatizing perceptions of TMD, and establish patient mutual aid communities to provide emotional support and a platform for sharing experiences (59). Simultaneously, efforts should be made to promote policy refinement (such as raising the proportion of medical insurance reimbursement) and construct an all-around rehabilitation environment for patients ranging from families to the community.

## 5 The review’s limitations

This study did not employ validated TMD-specific search strategies for literature retrieval (60). This limitation may have resulted in missed TMD-related qualitative studies and introduced selection bias during screening, thereby compromising the comprehensiveness of the included data and the reliability of the findings. Consequently, the study conclusions may exhibit a certain degree of bias. This study primarily integrates and analyzes existing qualitative research data. While covering multi-stage diagnostic and treatment experiences, it still fails to fully capture individual differences among patients with varying disease severity, ages, and geographic locations. Secondly, this study relies solely on patient perspectives to gather subjective health management needs, without conducting multi-perspective interviews with healthcare professionals and family caregivers. This approach may result in incomplete identification of specialized clinical needs and family support requirements. Future research should first conduct literature searches using validated TMD-specific search terms, collect multi-source data to enrich the journey map, and incorporate the perspectives of patients, caregivers, and medical professionals, thereby formulating more comprehensive and precise improvement strategies.

This study primarily integrates and analyzes existing qualitative research data. While covering multi-stage diagnostic and treatment experiences, it still fails to fully capture individual differences among patients with varying disease severity, ages, and geographic locations. Secondly, this study relies solely on patient perspectives to gather subjective health management needs, without conducting multi-perspective interviews with healthcare professionals and family caregivers. This approach may result in incomplete identification of specialized clinical needs and family support requirements.

Future research should first conduct literature searches using validated TMD-specific search terms, collect multi-source data to enrich the journey map, and incorporate the perspectives of patients, caregivers, and medical professionals, thereby formulating more comprehensive and precise improvement strategies.

## 6 Conclusion

This study developed a patient journey map based on systematically integrated qualitative evidence throughout the entire TMD patient journey. It illustrates the multidimensional experiences of TMD patients across their entire journey, providing evidence-based guidance for nursing practice. Findings suggest that nursing staff can play a crucial role in establishing a multidisciplinary team-based management support system to deliver comprehensive care, aid patients in emotional management, enhance their healthcare experience, and ultimately improve their quality of life.

This study developed a patient journey map based on systematically integrated qualitative evidence throughout the entire TMD patient journey. It illustrates the multidimensional experiences of TMD patients across their entire journey, providing evidence-based guidance for nursing practice. Findings suggest that nursing staff can play a crucial role in establishing a multidisciplinary team-based management support system to deliver comprehensive care, support patients’ emotional well-being, enhance their healthcare experience, and ultimately improve their quality of life.

## Data Availability

Publicly available datasets were analyzed in this study. This data can be found at: The data underlying the results presented in the study are available from the databases (access necessitates the utilization of the account of the affiliated institution, CNKI: https://www.cnki.net/ CBM: https://www.sinomed.ac.cn/index.jsp WanFang: https://www.wanfangdata.com.cn/ VIP: https://www.cqvip.com/ PubMed: https://pubmed.ncbi.nlm.nih.gov/ Embase: https://www.embase.com/landing?status=grey SCOUPS: https://www.elsevier.com/zh-tw/products/scopus/higher-education Cochrane Library: https://www.cochranelibrary.com/ Web of Science: https://access.clarivate.com/login?app=wos&alternative=true&shibShireURL=https:%2F%2Fwww.webofknowledge.com%2F%3Fauth%3DShibboleth&shibReturnURL=https:%2F%2Fwww.webofknowledge.com%2F&roaming=true PsyINFO: http://search.ebscohost.com/login.aspx?authtype=ip,uid&profile = ehost&defaultdb = psyh CINAHL: https://search.ebscohost.com/).
