# Supplementary material for: A patient journey map based on the experience of temporomandibular disorders patients: a qualitative systematic review and meta-synthesis
Source: Front Public Health. 2026 Feb 12;14:1769781. doi: 10.3389/fpubh.2026.1769781 (PMC12935881; doi:10.3389/fpubh.2026.1769781)
Supplement: Supplementary file 1 [file Supplementary_file_1.docx]

# 1.Embase session results (13 Mar 2025)

| No. | Query | Results |
| --- | --- | --- |
| #7 | #5 AND #6 | 47 |
| #6 | 'experience':ti,ab,kw OR 'psychology':ti,ab,kw OR 'feeling':ti,ab,kw OR 'emotion':ti,ab,kw OR 'challenge':ti,ab,kw OR 'life':ti,ab,kw OR 'needs':ti,ab,kw | 4477017 |
| #5 | #3 AND #4 | 194 |
| #4 | 'qualitative research':ti,ab,kw OR 'interview':ti,ab,kw OR 'focus groups':ti,ab,kw OR 'qualitative study':ti,ab,kw | 383287 |
| #3 | #1 OR #2 | 20817 |
| #2 | 'costen syndrome':ti,ab,kw OR 'craniomandibular disorders':ti,ab,kw OR 'craniomandibular joint syndrome':ti,ab,kw OR 'temporomandibular disorder':ti,ab,kw OR 'temporomandibular dysfunction':ti,ab,kw OR 'temporomandibular joint disease':ti,ab,kw OR 'temporomandibular joint diseases':ti,ab,kw OR 'temporomandibular joint disorders':ti,ab,kw OR 'temporomandibular joint dysfunction':ti,ab,kw OR 'temporomandibular joint dysfunction syndrome':ti,ab,kw OR 'temporomandibular joint pain':ti,ab,kw OR 'temporomandibular joint syndrome':ti,ab,kw OR 'temporomandibular joint disorder':ti,ab,kw | 7794 |
| #1 | 'temporomandibular joint disorder'/exp | 18339 |

2.**Pubmed：**

((((((experience[MeSH Terms]) OR (experience[Title/Abstract])) OR (psychology[Title/Abstract])) OR (emotion[Title/Abstract])) OR (feeling[Title/Abstract])) OR (needs[Title/Abstract])) AND (('Costen syndrome' OR 'craniomandibular disorders' OR 'craniomandibular joint syndrome' OR 'temporomandibular disorder' OR 'temporomandibular dysfunction' OR 'temporomandibular joint disease' OR 'temporomandibular joint diseases' OR 'temporomandibular joint disorders' OR 'temporomandibular joint dysfunction' OR 'temporomandibular joint dysfunction syndrome' OR 'temporomandibular joint pain' OR 'temporomandibular joint syndrome' OR 'temporomandibular joint disorder') AND (‘qualitative’ OR ‘mixed study’ OR ‘mixed research’ OR ‘mixed method’ OR ‘phenomenology∗’ OR ‘grounded theory’ OR ‘ethnography∗’ OR ‘case stud∗’ OR ‘action research’ OR ‘interview∗’ OR ‘focus group’ OR ‘observation∗’)) 231

**3.Web of science：**

((TS=('temporomandibular disorders' OR 'Costen syndrome' OR 'craniomandibular disorders' OR 'craniomandibular joint syndrome' OR 'temporomandibular disorder' OR 'temporomandibular dysfunction' OR 'temporomandibular joint disease' OR 'temporomandibular joint diseases' OR 'temporomandibular joint disorders' OR 'temporomandibular joint dysfunction' OR 'temporomandibular joint dysfunction syndrome' OR 'temporomandibular joint pain' OR 'temporomandibular joint syndrome' OR 'temporomandibular joint disorder')) AND TS=('experience' OR 'psychology' OR 'emotion' OR 'feeling' OR 'needs' OR 'emotion' OR 'challenge' OR 'life' OR 'needs')) AND TS=(qualitative’ OR ‘mixed study’ OR ‘mixed research’ OR ‘mixed method’ OR ‘phenomenology∗’ OR ‘grounded theory’ OR ‘ethnography∗’ OR ‘case stud∗’ OR ‘action research’ OR ‘interview∗’ OR ‘focus group’ OR ‘observation∗) 272

4.**CINAHL（EBSCOhost）：**

SU ( 'temporomandibular disorders' OR 'Costen syndrome' OR 'craniomandibular disorders' OR 'craniomandibular joint syndrome' OR 'temporomandibular disorder' OR 'temporomandibular dysfunction' OR 'temporomandibular joint disease' OR 'temporomandibular joint diseases' OR 'temporomandibular joint disorders' OR 'temporomandibular joint dysfunction' OR 'temporomandibular joint dysfunction syndrome' OR 'temporomandibular joint pain' OR 'temporomandibular joint syndrome' OR 'temporomandibular joint disorder' ) AND SU ( 'experience' OR 'psychology' OR 'emotion' OR 'feeling' OR 'needs' OR 'emotion' OR 'challenge' OR 'life' OR 'needs' ) AND SU ( qualitative’ OR ‘mixed study’ OR ‘mixed research’ OR ‘mixed method’ OR ‘phenomenology∗’ OR ‘grounded theory’ OR ‘ethnography∗’ OR ‘case stud∗’ OR ‘action research’ OR ‘interview∗’ OR ‘focus group’ OR ‘observation∗ ) 18

**5.PsyCINFO：**

SU ( temporomandibular joint disorder or tmj disorder or tmd or tmj dysfunction ) AND SU ( qualitative research or qualitative study or qualitative methods or interview ) AND SU ( experiences or perceptions or attitudes or views or feelings or qualitative or perspective ) 5

**6.Cochrane Library:**

#1 MeSH descriptor: [Temporomandibular Joint Disorders] explode all trees 1233

#2 ('experience' OR 'psychology' OR 'emotion' OR 'feeling' OR 'needs' OR 'emotion' OR 'challenge' OR 'life' OR 'needs'):ti,ab,kw 659378

#3 ('qualitative study' OR 'qualitative studies' OR 'qualitative research' OR 'interview' OR focus groups'):ti,ab,kw 103169

#4 ('Costen syndrome' OR 'craniomandibular disorders' OR 'craniomandibular joint syndrome' OR 'temporomandibular disorder' OR 'temporomandibular dysfunction' OR 'temporomandibular joint disease' OR 'temporomandibular joint diseases' OR 'temporomandibular joint disorders' OR 'temporomandibular joint dysfunction' OR 'temporomandibular joint dysfunction syndrome' OR 'temporomandibular joint pain' OR 'temporomandibular joint syndrome' OR 'temporomandibular joint disorder'):ti,ab,kw 2485

#5 #1 OR #4 2485

#6 #2 AND #3 AND #5 55

**7.SCOPUS:**

(TITLE-ABS-KEY("Temporomandibular Joint Disorders" or "temporomandibular disorders" ) AND TITLE-ABS-KEY ( ''experience' OR 'psychology' OR 'emotion' OR 'feeling' OR 'needs' OR 'emotion' OR 'challenge' OR 'life' OR 'needs' ) AND TITLE-ABS-KEY ( 'qualitative AND research' OR 'interview' OR 'focus AND groups' OR 'qualitative AND study' ) ) 10

**8.CNKI**：(“颞下颌关节紊乱病”/“颞下颌紊乱病”)和（心理/体验/感受/需求/经历）和（定性研究或质性研究或扎根理论或现象学或访谈或体验或感受）0

**9.VIP：**（题名或关键词=颞下颌关节紊乱病/颞下颌紊乱病） AND (题名或关键词=心理/体验/感受/需求/经历） AND （题名或关键词=定性研究或质性研究或扎根理论或现象学或访谈或体验或感受） 0

**10.Wan Fang：**（主题=颞下颌关节紊乱病/颞下颌紊乱病） AND (主题=心理/体验/感受/需求/经历） AND （主题=定性研究或质性研究或扎根理论或现象学或访谈或体验或感受） 0

**11.CBM**：颞下颌关节紊乱；颞下颌紊乱；体验；质性研究 0
