# Supplementary material for: A patient journey map based on the experience of temporomandibular disorders patients: a qualitative systematic review and meta-synthesis
Source: Front Public Health. 2026 Feb 12;14:1769781. doi: 10.3389/fpubh.2026.1769781 (PMC12935881; doi:10.3389/fpubh.2026.1769781)
Supplement: Supplementary file 2 [file Supplementary_file_2.docx]

| Synthesized findings | Type of research | Dependability | Credibility | ConQual score | Comments |
| --- | --- | --- | --- | --- | --- |
| Onset of symptoms and confusion in self-management | Qualitative research -phenomenological,grounded theory,descriptive - High | Downgrade one level Moderate* | Remains unchanged | Moderate** | The findings came from 14 papers |
|  |  |  |  |  | *Downgraded one level as the majority of studies (13 out of 14) scored 3 on questions related to the appropriateness of the conduct of the study |
|  |  |  |  |  | **Remains unchanged as all findings unequivocal |
| Diagnostic uncertainty and the dilemma of medical interaction | Qualitative research-phenomenological,grounded theory,descriptive - High | Downgrade one level Moderate* | Remains unchanged | Moderate | The findings came from 15 papers |
|  |  |  |  |  | *Downgraded one level as the majority of studies (14 out of 15) scored 3 on questions related to the appropriateness of the conduct of the study |
|  |  |  |  |  | **Remains unchanged as all findings unequivocal |
| The trade-offs and uncertainty of treatment options | Qualitative research-phenomenological,grounded theory,descriptive - High | Downgrade one level Moderate* | Remains unchanged | Moderate | The findings came from 14 papers |
|  |  |  |  |  | *Downgraded one level as the majority of studies (13 out of 14) scored 3 on questions related to the appropriateness of the conduct of the study |
|  |  |  |  |  | **Remains unchanged as all findings unequivocal |
| The long-term adaptation and the reconstruction of quality of life | Qualitative research-phenomenological,grounded theory,descriptive - High | Downgrade one level Moderate* | Remains unchanged | Moderate | The findings came from 14 papers |
|  |  |  |  |  | *Downgraded one level as the majority of studies (13 out of 14) scored 3 on questions related to the appropriateness of the conduct of the study |
|  |  |  |  |  | **Remains unchanged as all findings unequivocal |
