# Supplementary material for: A patient journey map based on the experience of temporomandibular disorders patients: a qualitative systematic review and meta-synthesis
Source: Front Public Health. 2026 Feb 12;14:1769781. doi: 10.3389/fpubh.2026.1769781 (PMC12935881; doi:10.3389/fpubh.2026.1769781)
Supplement: Supplementary file 3 [file Supplementary_file_3.docx]

| **Author**  **(s) &Publication Year** | **Research location** | **Name of data extractors/reviewers** | **Reason for exclusion** | **Reasons for inclusion** | **date of data extraction** |
| --- | --- | --- | --- | --- | --- |
| Garro et al. （1994） | USA | ShiNi Huang | / | The research subjects and content meet the inclusion criteria, and it is possible to extract the available data and content. | 2025/3/13 |
| Wolf et al(2006) | Sweden | Min Huang | The disease does not match. | / | 2025/3/13 |
| Wolf et al(2006) | Sweden | ShiNi Huang | The disease does not match. | / | 2025/3/13 |
| S. EITNER et al.(2009) | Germany | Min Huang | The disease does not match. | / | 2025/3/13 |
| Durham et al（2010） | UK | Min Huang | / | The research subjects and content meet the inclusion criteria, and it is possible to extract the available data and content. | 2025/3/13 |
| Vuckovic et al. (2010) | Portland | Min Huang | The research subjects were exposed to | / | 2025/3/13 |
| J. DURHAM et al. (2011) | UK | Min Huang | Insufficient data for mixed research | / | 2025/3/13 |
| J. DURHAM et al. (2011) | UK | ShiNi Huang | Insufficient data for mixed research | / | 2025/3/13 |
| S. Linsen, et al. (2012) | USA | ShiNi Huang | Post-TMD surgery | / | 2025/3/13 |
| Rollman et al.（2013） | Netherlands | ShiNi Huang | / | The research subjects and content meet the inclusion criteria, and it is possible to extract the available data and content. | 2025/3/13 |
| Akhter et al.(2013) | Japan | Min Huang | Insufficient qualitative data | / | 2025/3/13 |
| Briceñ o et al. (2013) | Colombia | Min Huang | Post-TMD surgery | / | 2025/3/13 |
| Au et al.(2014) | China | ShiNi Huang | Disease and age restrictions are met. | / | 2025/3/13 |
| Bonathan et al（2014） | UK | Min Huang | / | The research subjects and content meet the inclusion criteria, and it is possible to extract the available data and content. | 2025/3/13 |
| Mienna et al（2014） | Sweden | ShiNi Huang | / | The research subjects and content meet the inclusion criteria, and it is possible to extract the available data and content. | 2025/3/13 |
| Eaves et al（2014） | USA | ShiNi Huang | / | The research subjects and content meet the inclusion criteria, and it is possible to extract the available data and content. | 2025/3/13 |
| Sarah Peters et al.(2015) | UK | Min Huang | The disease does not match. | / | 2025/3/13 |
| Nilsson et al(2016) | Sweden | Min Huang | / | The research subjects and content meet the inclusion criteria, and it is possible to extract the available data and content. | 2025/3/13 |
| Alakailly et al. (2016) | USA | ShiNi Huang | Post-TMD surgery | / | 2025/3/13 |
| Gerbino et al. (2016) | Italy | Min Huang | Post-TMD surgery | / | 2025/3/13 |
| J. Kunjur et al. (2016) | UK | Min Huang | Post-TMD surgery | / | 2025/3/13 |
| Inglehart et al.(2017) | Japan | ShiNi Huang | The age of the research subject does not meet the requirements. | / | 2025/3/13 |
| Eaves et al（2017） | USA | ShiNi Huang | / | The research subjects and content meet the inclusion criteria, and it is possible to extract the available data and content. | 2025/3/13 |
| Desai et al. (2018) | South Africa | Min Huang | Post-TMD surgery | / | 2025/3/13 |
| Breckons et al (2017) | UK | Min Huang | The research subject is facial pain. | / |  |
| Elledge et al. (2018) | UK | ShiNi Huang | Post-TMD surgery | / | 2025/3/13 |
| Hazaveh et al.(2018) | Sweden | Min Huang | The disease does not match. | / | 2025/3/13 |
| Fjellman-Wlklund et al(2019) | Sweden | Min Huang | / | The research subjects and content meet the inclusion criteria, and it is possible to extract the available data and content. | 2025/3/13 |
| C. STORM MIENNA ET AL. (2019) | Sweden | Min Huang | Insufficient qualitative data | / | 2025/3/13 |
| M. Al-Baghdadi, et al. (2019) | UK | ShiNi Huang | Post-TMD surgery | / | 2025/3/13 |
| Ilgunas et al（2020） | Sweden | ShiNi Huang | The research subjects are dentists. | / | 2025/3/13 |
| Breckons et al (2020) | UK | Min Huang | The disease does not match. | / | 2025/3/13 |
| Ilgunas et al（2020） | Sweden | ShiNi Huang | / | The research subjects and content meet the inclusion criteria, and it is possible to extract the available data and content. | 2025/3/13 |
| Zumbrunn Wojczynska et al.(2020) | Switzerland | Min Huang | Post-TMD surgery | / | 2025/3/13 |
| B. Gupta et al. 2020 | Australia | ShiNi Huang | Post-TMD surgery | / | 2025/3/13 |
| Bijelic et al (2021) | Sweden | Min Huang | Internet therapy for teenagers | / | 2025/3/13 |
| Dinsdale et al（2021） | Australia | Min Huang | / | The research subjects and content meet the inclusion criteria, and it is possible to extract the available data and content. | 2025/3/13 |
| Dinsdale et al（2021） | Australia | ShiNi Huang | / | The research subjects and content meet the inclusion criteria, and it is possible to extract the available data and content. | 2025/3/13 |
| Taimeh et al（2022） | UK | Min Huang | / | The research subjects and content meet the inclusion criteria, and it is possible to extract the available data and content. | 2025/3/13 |
| H. A. VAN DER MEER ET AL. (2022) | Netherlands | Min Huang | Insufficient data for mixed research | / | 2025/3/13 |
| Grunberg et al (2022) | USA | Min Huang | The research subject is facial pain. | / | 2025/3/13 |
| Ilgunas et al（2023） | Sweden | ShiNi Huang | / | The research subjects and content meet the inclusion criteria, and it is possible to extract the available data and content. | 2025/3/13 |
| E. Elstad et al. (2023) | USA | Min Huang | Insufficient data for mixed research | / | 2025/3/13 |
| Taimeh et al (2023) | UK | ShiNi Huang | Summary | / | 2025/3/13 |
| Penlington et al（2024） | UK | Min Huang | / | The research subjects and content meet the inclusion criteria, and it is possible to extract the available data and content. | 2025/3/13 |
| Baggen et al（2024） | Netherlands | ShiNi Huang | / | The research subjects and content meet the inclusion criteria, and it is possible to extract the available data and content. | 2025/3/13 |
| Olsson et al（2024） | Sweden | Min Huang | / | The research subjects and content meet the inclusion criteria, and it is possible to extract the available data and content. | 2025/3/13 |
| Safour et al（2024） | Canada | ShiNi Huang | / | The research subjects and content meet the inclusion criteria, and it is possible to extract the available data and content. | 2025/3/13 |
| Taqi et al. (2024) | Pakistan | Min Huang | The research subjects are dentists. | / | 2025/3/13 |
| FABRIZIA et al (2024) | Italy | ShiNi Huang | Summary | / | 2025/3/13 |
| H.A. VANDERMEER et al. (2024) | Netherlands | Min Huang | Insufficient data for mixed research | / | 2025/3/13 |
